# *Jalaneti* (saline nasal irrigation) as primary intervention in suspected rhino-orbito-cerebral mucormycosis helps improving the recovery: A case report

**DOI:** 10.1016/j.jaim.2021.08.009

**Published:** 2021-11-01

**Authors:** Sanjeev Rastogi, Ankita Verma

**Affiliations:** PG Department of Kaya Chikitsa, State Ayurvedic College and Hospital, Lucknow, 226003, India

**Keywords:** Mucormycosis, *Jalaneti*, Saline nasal irrigation, Case report, Diabetes

## Abstract

The ongoing COVID-19 pandemic has resulted in several opportunistic infections like mucormycosis (MCR) to surface. Although this is commonly afflicting immunocompromised people managed through prolonged ICU care, epidemiological observations suggest that it is also associated with conditions like uncontrolled diabetes. Due to its invasive nature and systemic reach, MCR has high mortality warranting an early diagnosis and treatment. We present here a case of a non-COVID, diabetic patient having acute onset paranasal and periorbital swelling with headache suspected for rhino-orbito-cerebral MCR. The case was innovatively dealt with *jalaneti* (saline nasal irrigation) seeing a delay in the institution of definitive anti-fungal therapy. Six sittings of *jalaneti* in four days had been able to give near complete symptomatic relief in paranasal swelling and headache even before the endoscopic nasal debridement and anti-fungal therapy was initiated.

Seeing the urgency of diagnosis and treatment in any suspected case of MCR, a simple and self-administrable procedure like *jalaneti* seems to have a high value for its possible role in reducing the sinus inflammation and reducing the disease intensity in order to find more time for the proper diagnosis and treatment initiation. Negligible cost of *jalaneti*, its easy administration, and minimal adversity potential are additional advantages for proposing *jalaneti* as a possible prophylaxis in MCR. More serious clinical research is urgently required to confirm the observations made in this single case report and to extend its benefits to the people suffering with MCR.

## 1 Introduction

As the coronavirus disease 2019 (COVID-19) pandemic is evolving, more complications associated with COVID-19 are emerging. After being massively struck by the second wave of the pandemic, in India mucormycosis (MCR) emerged as a potential threat to COVID sufferers who had recovered from severe state of the disease. Although MCR had been prevalent in the country since past, its current trend in the pandemic particularly, during the second wave has been alarming. India has reported 40,854 cases of MCR so far from 28 states/UTs in the country. Among all MCR cases reported in India, 85.5 per cent cases had a history of COVID-19 and 64.11 per cent had diabetes [1]. India reports highest prevalence (140 cases per million population) of MCR in the world [2] and accounts for 71% of total cases reported globally [3]. With diabetes mellitus (DM) emerging as a single-most common risk factor for developing MCR, India with its second-largest adult population (aged 20–79 years) with DM serves as a potential niche for MCR explosion. Current epidemiological trend of MCR in India during the pandemic is similar to what is observed in a recent nationwide multi-center study on MCR in India showing 57% of MCR patients having uncontrolled diabetes mellitus and 18% having diabetic ketoacidosis [4]. Besides uncontrolled DM, other predisposing factors for MCR are immunosuppression by steroids, prolonged ICU stay, and co-morbidities like post-transplant/malignancy and voriconazole therapy. Its association with COVID-19 is proposed on preclude of unwarranted steroid therapy and prolonged ICU stay along with oxygen therapy. Indian pandemic epidemiology of MCR is suggestive of severe COVID-19 with uncontrolled DM as a deadly combination. MCR is an angio-invasive fungal infection, associated with high morbidity and mortality even after active management. Rhino-orbito-cerebral presentation associated with uncontrolled diabetes is the predominant category of its presentation in the Indian subcontinent. The causative agents of MCR vary across different geographic locations. *Rhizopus arrhizus* is the most common agent isolated worldwide. *Apophysomyces variabilis* is predominant in Asia and Lichtheimia species in Europe. The new causative agents, *R. homothallicus*, *Mucor irregularis*, and *Thamnostylum lucknowense* are also occasionally reported from Asia.

MCR has a poor prognosis with high mortality rate of 17–51% [5]. Mortality is higher in cases where the diagnosis is delayed. Surgical debridement along with anti-fungal treatment is the conventional approach to deal with MCR [5]. Recently, Indian Council for Medical Research has issued an evidence-based advisory for screening, diagnosis, and management of MCR [6]. MCR poses substantial challenges in its diagnosis and management [7]. Clinical approach to diagnose MCR lacks sensitivity and specificity. In rhino-sinus MCR, CT is the investigation of choice to study the invasion of bone and soft tissue and extension of it to the central nervous system. MRI is more sensitive than CT for the investigation of possible cerebrovascular complications. Microscopy (direct and on histopathology) and culture are the cornerstones of diagnosis. Molecular assays can be used either for detection or identification of mucormycetes, and they can be recommended as complement to the conventional diagnostic procedures.

During the COVID-19 pandemic, resurgence of MCR has emerged as a great threat to those who could survive the severe SARS-CoV-2 infection by having a rigorous and intensive management. This is noticed that many of such survivors have developed diabetes as a sequel of the main disease owing to the intensive steroid therapy, sustained stress, and suspected pancreatic injury. This might have increased the possibility of MCR association with COVID–19 survivors. Seeing its re-emergence during the pandemic and finding its association with COVID-19, many states in India have notified it under the Epidemic Diseases Act (EDA) 1897. This provision has made any occurrence of MCR essential to be notified and to be treated as per standard guidelines [8].

Considering its notification under the EDA 1897 and seeing its rapidly progressive invasive course with high morbidity, Ayurveda and other AYUSH systems refrained themselves from getting involved in its treatment. Although seeing the adjuvant possibility of Ayurveda in comprehensive management of MCR, a few states have allowed using selected Ayurveda medicines as adjuvant therapy in MCR cases being treated at modern health care facilities [9]. Outcomes of such novel integrative approaches are yet to be seen.

Rapid re-emergence of MCR in India in the backdrop of exceptionally high rate of SARS-CoV-2 infection in the country left behind a large population in the post-COVID convalescence phase. This, when added with a large population of long-standing uncontrolled DM, makes the situation highly worrying. Exceptionally high mortality of the disease, invasiveness of the pathogen causing extensive tissue and organ damage, general sanitation conditions, poor urban waste disposal mechanisms and high humid and warm climatic conditions in India, makes it a most suitable nursery for growth and sustenance of fungal infections. Early diagnosis and treatment are the key determinants deciding the net outcome in a given MCR case. We however, see that both of these are often delayed and hence, a vital time is lost. In this situation, initiation of some simple procedure like *jalaneti* in all suspected cases till a definitive diagnosis is arrived may prevent many complications.

We present here a case of a non-COVID, uncontrolled diabetic patient having acute onset paranasal and periorbital swelling with headache. After initial suspicion of having MCR through paranasal MRI, the confirmation of diagnosis and initiation of anti-fungal therapy was delayed for 4 days. This delay was dealt with a simple lukewarm saline nasal irrigation called *jalaneti* upon the recommendation of treating physician (SR). The process was done altogether 6 times in a span of 4 days till the diagnostic nasal endoscopy was conducted. *Jalaneti* in this case, alone in the absence of any specific anti-fungal therapy, was found to be able to give almost complete relief from periorbital and paranasal swelling and headache. Diagnostic nasal endoscopy done after 4 days of initiation of *jalaneti* could not reveal any noticeable pathology in the nasal cavity or sinuses. It could not reveal any fungal element in it either.

Despite failure in establishing a definitive diagnosis, seeing the high suspicion of the case for it being MCR, the national protocol for MCR management was followed. Surgical debridement was attempted subsequently and specific anti-fungal therapy was initiated. The patient was discharged from the hospital after 2 weeks having no remaining symptoms.

This case places before us a highly plausible hypothesis of possible modification of the course of rhino—orbito-cerebral MCR through saline nasal irrigation done in the spirit as described in Yoga texts. In case of diagnostic and therapeutic delay as is commonly seen in such cases, this simple procedure may prove to be a life savior. Benefits of *jalaneti* in cases of sinusitis have been extensively studied and experimented globally; however, its application in MCR has never been reported. This case proposes to strengthen the standard treatment protocol of MCR by initiating *jalaneti* in cases suspected for MCR and where a confirmed diagnosis is yet to arrive. More evidences in the form of case series, pilot studies, and clinical trials are however warranted to establish any specific benefits associated with *jalaneti* in cases of rhino-orbito-cerebral MCR.

## 2 Case description

This case report is about a 64 year-old average built, diabetic (history of 12 years, largely uncontrolled) male having an acute onset of periorbital and paranasal swelling along with headache (18.5.2021). The onset was preceded by his working in home garden unprotected and handling the compost/cow dung manure for long hours. It was suspected as a case of rhino-orbito-cerebral MCR on the basis of clinical history and MRI-PNS findings (19.5.2021, Table 1). A surgical debridement was recommended; however, it was delayed for 4 days due to the bed unavailability in MCR ward at a secondary care teaching hospital in Lucknow having a MCR specialty unit. The patient was suggested to start *jalaneti* with lukewarm saline water twice a day as an alternative advice till, a definitive treatment was sought as per the standard treatment protocol. To ease the process of *jalaneti* and to ensure the isotonic concentration and sterile nature of salt solution, the patient was recommended to use 100 ml squeezable plastic bottle of normal saline warmed through a water bath. The *neti* was introduced in a partially tilted position of head by putting the nozzle of the bottle in the nostril and squeezing the bottle with light pressure. After doing the process in one nostril with using about 50 ml solution, rest half solution was used through other nostril in the same way. The process was well-tolerated and was without any noticeable discomfort. The process was repeated 6 times in 4 days (from 19.5.21 to 22.5.21) till he was admitted in the MCR ward at the designated hospital (21.5.2021) and had undergone a diagnostic nasal endoscopy (23.5.2021).

**Table 1:** Findings of contrast-enhanced MRI brain, orbit, and paranasal sinuses (19.05.2021).

• Large peripherally enhancing and expansible collection in the frontal sinus (on the right of the midline) extending to the right anterior ethmoidal sinus likely infective (pyocele).
• Sinusitis involving the bilateral maxillary and ethmoidal sinuses with mucosal thickening in the sphenoid sinus.
• Diffuse inflammatory changes involving the subcutaneous tissue of both sides of right side of face and bilateral masticator space with enhancing soft tissue collection in left paranasal region. - Likely inflammatory/post infective etiology (secondary spread of sinus infection).
• Diffuse cerebral and cerebellar atrophy with old lacunar infarcts in the left thalamus and bilateral cerebellar hemisphere.
• Partial empty sella.

Diagnostic endoscopy of paranasal sinuses however, did not reveal any specific finding. KOH mount for fungal elements done on 23.5.2021 came negative. Seeing a high probability of MCR on the basis of clinical history and MRI findings, standard anti-fungal therapy was subsequently instituted as per the MCR protocol. Nasal packing was removed on 24.5.2021. Redness and swelling around the left eye was noticed next day (25.5.2021) for which antibiotics were started. During his hospital stay also, the patient was recommended to do nasal douching with alkaline saline water. The patient remained admitted in the hospital till next week and was subsequently discharged on 3.6.2021. Major events during the course of illness and treatment are summarized as Table 2. He started doing *jalaneti* again, once a day, after the discharge from the hospital. Till the date of writing this case report, he is perfectly fine and does not have any traces of earlier symptoms related to MCR.

**Table 2:** Timeline for major events related to case.

Date	Major Event
18.5.2021	Slight swelling observed around left eye and left side of nose.
19.5.2021	Marked swelling and redness around both eyes and left side of nose. MRI PNS done. Finding were suggestive of MCR Rapid Antigen Test for COVID-19 was negative
20.5.2021	*Jalaneti* started
21.5.2021	RT-PCR test was negative *Jalaneti* done two times Swelling around eyes and nose substantially reduced Patient admitted in MCR ward, KGMU, Lucknow
22.5.2021	*Jalaneti* continued two times in a day KOH mount was done for fungal test
23.5.2021	*Jalaneti* done once Diagnostic nasal endoscopy done. Nasal packing done. Amphotericin B started
24.5.2021	Nasal packing was removed
25.5.2021	Redness and swelling in left conjunctiva was noticed. Fungal element report came negative.
26.5.2021–31.5.2021	Amphotericin B continued
3.6.2021	Discharged from the hospital

## 3 Discussion

*Jalaneti* is a simple nasal lavage technique using lukewarm isotonic saline water to clean the nasal passage. Originating from the texts of Yoga and having a reference to *shatkarma* (six procedures), *neti* is one among 6 cleaning procedures recommended to prepare the body and mind for higher stages of Yoga [10]. In recent years, *jalaneti* has received sufficient attention of physicians, ENT specialists, and veterinarians for its beneficial effects in cases of acute and chronic sinusitis, nasal polyp, upper respiratory tract infections and sino-nasal aspergillosis [[11], [12], [13], [14], [15], [16], [17]]. At least two Cochrane Database Systematic Reviews conducted to explore the evidence level of saline nasal irrigation in acute upper respiratory tract infection [18] and chronic sinusitis [19] have concluded about the possible benefits of nasal saline irrigation in given conditions. However, they have also warranted about the need of trials involving larger numbers of participants and reporting of standardized and clinically meaningful outcome measures. *Jalaneti* is conventionally done with the help of a metallic, plastic or porcelain *neti* pot having a nozzle for pouring water and the saline solution is conventionally made by mixing one teaspoon table-salt in the pot filled with drinkable lukewarm water. The process, despite of its therapeutic benefits had numerous feasibility problems. A few notable practical problems are sterilization of pot, water, salt, and maintaining the temperature and essential pressure allowing the solution to reach deeper inside the nasal cavity. In conventional ENT practice, this is done by use of a syringe to push the solution inside. The reach of saline solution to the openings of sphenoid, ethmoid, and frontal sinuses located at the top of the nasal cavity was however doubted and proposed that the irrigation may reach these openings only when the head is positioned upside down [20]. To ease out this problem, conventional *jalaneti* was proposed to be done in a specific sitting posture (*kagasana*) having the head tilted to the opposite side of the nostril to be irrigated. To overcome all such procedural hiccoughs related with *jalaneti*, Rastogi et al. proposed a novel method of doing it with a squeezable plastic normal saline bottle of 100 ml used for intravenous infusion [21]. This method had the novelty of having pre-sterilized, pre-measured isotonic saline solution in a squeezable bottle where the pressure to push the solution inside the nasal cavity could have been easily generated by the patient himself thus, allowing it to reach deep in the sinuses with much ease.

*Jalaneti* has a beneficial role in sinusitis, probably due to its cleaning action which may be attributed to its warmth and reach to the deeper sinuses where conventionally drugs are unable to reach. In cases of fungal sinus infections, it may have additional benefits offered through saline solution being non-promotive to fungus growth. This is a customary practice in fishery to add salt in water to prevent topical fungal growth on the skin of fish [22]. Salt solutions in various concentrations have also been used in farming to prevent plant species from fungal infections [23]. Its role in topical or systemic fungal infections in humans however, is not yet explored. This case report, therefore provides an important lead for possible exploration in this direction.

As it is shown in the case studied, an early institution of *jalaneti* in the modified format as is used in the case seems to have plausible possibilities of halting the growth of fungus in cases of rhino-orbito-cerebral MCR. The simplest thing it might offer is to lavage the sinuses thereby, diluting the concentration of available fungal mycelia and spores. Moreover, by providing a milieu interior un-favoring the growth of fungus, it may prevent further growth of whatever little amount of fungus still remaining. Supplemented with standard anti-fungal intervention, this integrative management may offer a quick and complete resolution of a most deadly disease which is keeping India on its toes during the pandemic. Another important dimension of the case and used intervention is its minimal cost. Conventionally, treating MCR is found to be very expansive and many patients are unable to bear the high cost of such treatment. Contrary to this, *jalaneti* has negligible cost added with immense benefits. Saline nasal irrigation is found to be completely safe and nowhere in the world has any adversity related to this been reported so far.

It is also suggested that Ayurvedic innate constitution (*prakriti*) may possibly help selecting the right people for this intervention in order to ensure maximum benefits. It may be much beneficial in *pitta* people as compared to *vata* or *kapha* dominant people. People having allergic inflammatory sinus disorders do not benefit much from saline nasal irrigation. Traditionally, such nasal applications are also not advisable among children. This general caution is recommended to be utilized while using *jalaneti* in clinical practice.

Despite of highly encouraging results, the case however, had limitation about its diagnostic certainty. For a suspicious case of MCR on the basis of clinical history, tissue biopsy and CT scan should have been performed. Somehow, despite treating the case as a definitive case of MCR and following standard MCR treatment protocol, these investigations could not be performed. Had they been performed, they surely would have added additional value to the reported case.

## 4 Conclusion

During ongoing COVID-19 pandemic, MCR widely known as black fungus infection re-emerged in concerning proportion. India currently holds the highest number of MCR cases reported in the world so far. Rapidly instituting morbidity, tissue and organ invasion by the pathogen and high mortality associated with MCR are worrying factors associated with pathogenesis. High cost of the conventional anti-fungal therapy, delay in diagnosis and institution of therapy and high mortality despite of adequate treatment are further reasons of concern. In this scenario, a simple provision of *jalaneti* or saline nasal irrigation in a probable case of rhino-orbito-cerbral MCR leading to near complete clinical recovery before the standard anti-fungal therapy was actually instituted, raises the hope for some pragmatic solution to halt the progress of the pathogenesis and to reduce its intensity. In case of any delay in diagnosis or initiation of standard anti-fungal therapy in suspected MCR cases, this low cost intervention is capable of buying time for more definitive therapies and in milder cases may suffice the treatment. Observations made in this single case of MCR treated with *jalaneti* are sensitive enough to initiate serious clinical trials in this area without any further delay.

## 5 Patient's perspective

First of all thanks to all the doctors who helped me in my time of need. When I first got my symptoms of facial swelling and all, the ENT specialist I consulted had recommended for MRI. After seeing the MRI, I was diagnosed as a black fungus case and was advised for immediate surgery. My family was worried and sought a second opinion from Ayurveda. I was advised for doing *jalaneti* till more definitive therapies were started. For me, nasal blockage, running nose, and headache were the major problems as they were not allowing me to sleep properly. However, after doing *jalaneti* for 2nd and 3rd time, I had much relief. It was a simple cleansing process. I could do it myself. Thank you for your simple and free prescription of *jalaneti* which costs almost nothing yet helped a lot.

## 6 Informed consent

Informed consent was obtained from the patient regarding the initiation of *jalaneti* intervention. Consent has also been obtained for publication of this case report for the purpose of dissemination of knowledge among medical fraternity.

## Source of funding

None.

## Conflict of interest

None.

## Author contributions

**Sanjeev Rastogi**: Conceptualization, Methodology, Original draft preparation, Supervision. **Ankita Verma**: Investigation, Resources, Writing - Review and editing.
